# *Yersinia enterocolitica palearctica *serobiotype O:3/4 - a successful group of emerging zoonotic pathogens

**DOI:** 10.1186/1471-2164-12-348

**Published:** 2011-07-06

**Authors:** Julia Batzilla, Uladzimir Antonenka, Dirk Höper, Jürgen Heesemann, Alexander Rakin

**Affiliations:** 1 Max von Pettenkofer-Institute, LMU, Munich, Germany; 2 Institute of Diagnostic Virology, Friedrich-Loeffler-Institute, Greifswald-Insel Riems, Germany

## Abstract

**Background:**

High-pathogenic *Y. enterocolitica *ssp. *enterocolitica *caused several human outbreaks in Northern America. In contrast, low pathogenic *Y. enterocolitica *ssp. *palearctica *serobiotype O:3/4 is responsible for sporadic cases worldwide with asymptomatic pigs being the main source of infection. Genomes of three *Y. enterocolitica *ssp. *palearctica *serobiotype O:3/4 human isolates (including the completely sequenced Y11 German DSMZ type strain) were compared to the high-pathogenic *Y. enterocolitica *ssp. *enterocolitica *8081 O:8/1B to address the peculiarities of the O:3/4 group.

**Results:**

Most high-pathogenicity-associated determinants of *Y. enterocolitica *ssp. *enterocolitica *(like the High-Pathogenicity Island, *yts1 *type 2 and *ysa *type 3 secretion systems) are absent in *Y. enterocolitica *ssp. *palearctica *serobiotype O:3/4 genomes. On the other hand they possess alternative putative virulence and fitness factors, such as a different *ysp *type 3 secretion system, an RtxA-like and insecticidal toxins, and a N-acetyl-galactosamine (GalNAc) PTS system (*aga*-operon). Horizontal acquisition of two prophages and a tRNA-Asn-associated GIYep-01 genomic island might also influence the *Y. enterocolitica *ssp. *palearctica *serobiotype O:3/4 pathoadaptation. We demonstrated recombination activity of the PhiYep-3 prophage and the GIYep-01 island and the ability of the *aga*-operon to support the growth of the *Y. enterocolitica *ssp. *enterocolitica *O:8/1B on GalNAc.

**Conclusions:**

*Y. enterocolitica *ssp. *palearctica *serobiotype O:3/4 experienced a shift to an alternative patchwork of virulence and fitness determinants that might play a significant role in its host pathoadaptation and successful worldwide dissemination.

## Background

The gram-negative bacterium *Yersinia enterocolitica *is a widely disseminated gastrointestinal pathogen that belongs to the genus *Yersinia *together with enteropathogenic *Y. pseudotuberculosis *and the plague agent, *Y. pestis*. It has been proposed that *Y. enterocolitica *and *Y. pseudotuberculosis *have diverged within the last 200 million years while *Y. pestis *is a more recent descendant of *Y. pseudotuberculosis *[1,2]. All these species have evolved with diverse clinical symptoms. *Y. pseudotuberculosis *can cause tuberculosis-like symptoms in animals. In humans, the clinical manifestations are similar, but often they are more severe compared to those with *Y. enterocolitica*. *Y. pseudotuberculosis *infections can mimic appendicitis, mainly in children, and have similar extra intestinal sequelae compared to *Y. enterocolitica*. *Y. pestis *is the agent of the plague, transmitted by the bite of an infected flea and it is primarily a rodent pathogen.

*Yersinia enterocolitica *can be differentiated by bio- and serotyping [3,4]. Biotype (BT) 1A strains are considered as non-pathogenic, whereas high-pathogenic BT1B (predominant in the U.S.A.) and low to moderate-pathogenic BT2-5 (predominant in Europe, Asia and Australia) are enteropathogenic for humans and animals. *Y. enterocolitica *serogroup O:3 biotype 4 (in the following designated as serobiotype O:3/4 or O:3/4) comprises about 80-90% of human isolates in Germany and Europe, with rising global relevance [5-7]. It is responsible for gastroenteritis, lymphadenitis and various extra intestinal sequelae as erythema nodosum and reactive arthritis [8]. Asymptomatic and ill pigs are the main animal reservoir of this serobiotype [9,10], leading to a high submission of O:3/4 contaminations in butcher shops in Germany and countries in north-eastern Europe [11-14]. In contrast, *Y. enterocolitica *biotype 1B strains (also called New World strains) were documented predominantly in the U.S.A. as human outbreak and environmental isolates. Pathogenicity analysis, however, has been mainly focused on *Y. enterocolitica *serobiotype O:8/1B, of which a complete genome sequence is available (strain 8081, [15], accession no. NC_008800.1 and NC_008791 (plasmid)). The differentiation between biotype 1B and the Old World strains has been legitimated in the assembly of two subspecies, *Y. enterocolitica *ssp. *enterocolitica *for biotype 1B and *Y. enterocolitica *ssp. *palearctica *for the Old World strains [16].

To compare these two groups of geographically and phylogenetically distinct yersiniae, we determined the complete genome sequence of the European serobiotype O:3/4 DSMZ reference strain Y11 isolated from a patient stool (EMBL accession numbers: FR729477 and FR745874 (plasmid) as announced recently [17]) and compared it with the available *Y. enterocolitica *ssp. *enterocolitica *8081 O:8/1B genome. Draft sequences of two other *Y. enterocolitica *ssp. *palearctica *O:3/4 strains of human origin, named Y8265 and Y5307 (derived from a human patient isolate from France and an arthritis positive human patient isolate, respectively) and a closely related *Y. enterocolitica *ssp. *palearctica *strain (named Y5,27P) of serobiotype O:5,27/3 were used when appropriate to gain a better insight into peculiarities of the *Y. enterocolitica *ssp. *palearctica*. Comparison of these closely related pathogens enables us to uncover potential pathogenicity and fitness determinants involved in pathoadaptation and worldwide dissemination of *Y. enterocolitica *ssp. *palearctica *O:3/4.

## Results

### 1. General characteristics

The genome of *Y. enterocolitica *ssp. *palearctica *Y11 (serobiotype O:3/4) consists of a circular chromosome spanning 4,553,420 bp with a GC content of 47.01% and a virulence associated pYVO3 plasmid of 72,460 bp with an overall GC content of 43.99% (see Table 1 and additional file 1). The tabular SEED comparison between serobiotype O:3/4 strains Y11, Y8265, Y5307, serobiotype O:5,27/3 and serobiotype O:8/1B strain 8081 is added as additional file 2.

**Table 1:** General features of the genome and virulence plasmid of serobiotypes O:3/4 strain Y11 and O:8/1B strain 8081.

Feature	Genome Y11	Genome 8081	Plasmid pYVO3 Y11	Plasmid pYVO8 8081
**Genome size**	4553420	4615899	72460	67721

**GC content in %**	47.01	47.27	43.99	43

**Number of coding sequences (CDS)**	4355	3978	112*	72

**Average size of CDS**	884	968	506	672

**Coding density in %**	85	83	78	71

**rRNA clusters**	7	7	none	none

**tRNAs**	70	81	none	none

*) The higher number of CDS for the O:3/4 pYV plasmid compared to O:8/1B is evoked by more transposase-like CDS (including also remnant or fragmented transposases) and more than 20 hypothetical CDS with no strong similarity on protein level to serobiotype O:8/1B. In addition, we found an *arsABRH *gene cluster present in the pYV plasmid of serobiotypes O:3/4 and O:9/2, but not in O:8/1B.

### 2. *Y. enterocolitica *ssp. *enterocolitica *genes absent from *Y. enterocolitica *ssp. *palearctica*

Certain virulence determinants associated with the high-pathogenicity phenotype of *Y. enterocolitica *ssp. *enterocolitica *are missing in serobiotype O:3/4. The most prominent are the High-Pathogenicity Island (HPI), involved in the yersiniabactin-mediated iron uptake [18-20], and two chromosomally encoded secretion systems, *ysa *T3SS and *yts1 *T2SS, located within the Plasticity Zone (PZ) [21]. Besides HPI and PZ several genomic islands (YGI-2 and YGI-3) and prophages (e.g. ΦYE250, ΦYE185 (encoding the Vaps virulence-associated proteins)) described for serobiotype O:8/1B strain 8081 are absent in O:3/4. In addition, the pYV plasmids of O:3/4 and O:8/1B are more divergent than the corresponding genome sequences. In contrast, the pYVe227 plasmid of serobiotype O:9/2 (accession no. AF102990) is closely related to pYVO3 plasmid (additional file 3). Also the clusters for the LPS biosynthesis differ. In O:3/4, two separate clusters are present as reported previously (for the outer core, Y11_19901-20011 and O-antigen, Y11_16711-16781) [22,23].

Most of the genome differences between O:8/1B and other serobiotypes have been described previously [15]. Nevertheless there are some additional potential virulence-associated genes that have been uncovered by our genome comparison, including putative haemolysins (YE2407-2408, YE2966), putative adhesins (YE1873) and a putative *ospG*-like gene (YE3860).

### 3. *Y. enterocolitica *ssp. *palearctica *O:3/4 specific genes absent from *Y. enterocolitica *ssp. *enterocolitica *O:8/1B

#### Putative toxins

**Figure 1:**
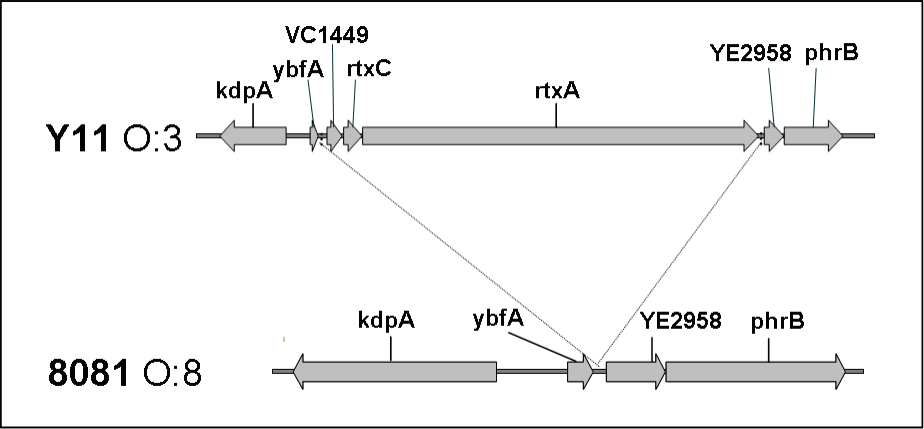
**RtxA toxin cluster in *Y. enterocolitica *ssp. *palearctica *strain Y11 (O:3/4)**. This gene cluster is inserted into the backbone of *Y. enterocolitica *ssp. *enterocolitica *strain 8081 (O:8). The cluster comprises 10.6 kbp encoding three genes, *rtxA*, *rtxC *and a hypothetical VC1449 gene (according to a protein homolog of *Vibrio cholerae*).

In serobiotype O:3/4 we found a gene cluster encoding an RtxA-like protein and its adjacent genes, *rtxC *and *rtxH*, (Y11_18761, Y11_18771 and Y11_18781) (see Figure 1). The large RtxA protein (Y11_18761, about 350 kDa) could play a role in the pathogenesis of O:3/4, since members of the wide family of Rtx proteins have been shown to be major contributors to pathogenicity [24-28]. The effector domains in the middle of the protein show no homology to already known family members. Therefore, the potential mode of action of the RtxA-like protein is unclear in this strain. In addition, the genes encoding the RtxA secretion system (Y11_10141-10171) are interrupted by a frame shift in O:3/4, raising the question of functional RtxA export.

Bacteriocins constitute a large group of bacterial toxins used to inhibit growth of closely related bacteria. The bacteriocin Y11_33511 in O:3/4 shows sequence homology to pyocin-like proteins and DNAses, therefore being a potential endonuclease enzyme. It is followed by two putative immunity proteins (Y11_33521-33531). The duplication of immunity protein like genes could hint at a particular toxin with extreme toxicity for its host.

A member of cell wall-associated hydrolases (Y11_03361, a putative invasion-associated protein) of about 422 amino acids is encoded in a 2 kbp large genomic region absent from O:8/1B strain 8081. The hydrolase encoding CDS is located between *yeiH *and *yeiE*, encoding a potential membrane protein and a transcriptional regulator with yet unknown functions. A 3,075 bp putative invasion precursor gene (Y11_38661) and a *hlyD *like gene (Y11_09551) have been found in *Y. enterocolitica *O:3/4. The putative invasin revealed homology to Ig-like domains and has a similar domain structure typical of invasion proteins. Genes of the ABC transporter family ([29,30]) lie adjacent to *hlyD *in O:3/4 that support its possible export.

We also found several toxin-antitoxin systems in serobiotype O:3/4 that are absent or different from those found in serobiotype O:8/1B. One toxin-antitoxin system (TA) was annotated as YgiT-(antitoxin, Y11_40161) and YgiU-(toxin, Y11_40151) like proteins in O:3/4. Kasari *et al*. reported that the protein YgiU inhibits growth and induces rapid shutdown of protein synthesis *in vivo*. The cluster is transcriptionally repressed by YgiT and activated by HipA [31]. Another TA cluster found in O:3/4 is annotated as YfjZ (antitoxin, Y11_30951) and YpjF (toxin, Y11_30941), reported to be a putative part of a defective prophage with unknown function [32].

#### Insecticidal toxin cluster

**Figure 2:**
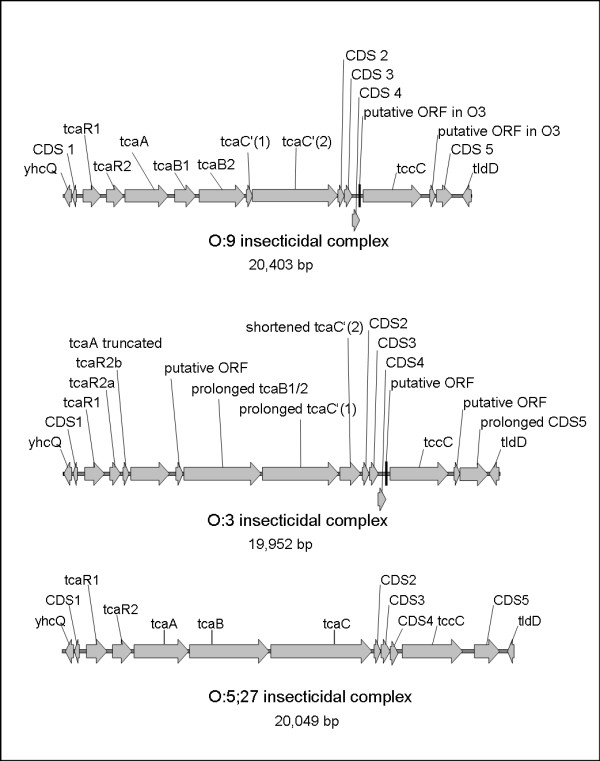
**Insecticidal toxin gene complex in *Y. enterocolitica *O:9/2, O:3/4 and O:5,27/3**. The structural organisation of the cluster is the same in all serobiotypes, but the encoded proteins differ in length and are to some extent fragmented. For O:5,27/3, the cluster is the only one with only a single *tcaC *gene. In addition, the *tcaR2 *is, as well as *tcaB*, complete.

An insecticidal toxin cluster (Tc, Y11_26921-Y11_27061), initially described in serobiotype O:9/2 [33], is found in serobiotype O:3/4 downstream of *tldD*, Y11_27071. The toxin cluster comprises four proteins, Tca, Tcb, Tcc and Tcd and is represented in serobiotype O:9/2 by TcaA, TcaB, TcaC and TccC. It was shown that these toxins are active at temperatures below 30°C and the lysates could kill *M. sexta *within 5 days [34]. Interestingly, TcaB is split into two proteins and *tcaC *is interrupted by a stop codon. Still the system was proven to be functional in O:9/2. In serobiotype O:3/4 one of the two putative *tcaA *regulatory genes is split into two smaller ones and TcaA (Y11_26951) is a truncated version of the serobiotype O:9/2 protein (see Figure 2). TcaB (Y11_26971) is a single protein in contrast to O:9/2, and the *tcaC'(1) *gene (Y11_26981) undergoes a frame shift at position 3498 (not at position 314 as in O:9/2). Since this gene cluster differed surprisingly between strains of the same subspecies, we analysed the Tc cluster of the related serobiotype O:5,27/3. This strain also harbours an insecticidal toxin complex closely related to that of serobiotypes O:3/4 and O:9/2. The cluster of O:5,27/3 is the only one with a single *tcaC *gene (Figure 2). In addition, the *tcaR2 *is, as well as *tcaB*, not fragmented. Another putative insecticidal toxin encoding CDS (Y11_05031), which is not clustered with the Tc cluster described above, is present in O:3/4. The protein is homologous to the insecticidal TccC2 and TccC3 proteins of *Y. enterocolitica *O:9/2.

#### Flagellar genes, beta-fimbrial genes and other fimbriae

A large 21 kbp flagellar cluster (Y11_24071-24361) present in O:3/4 genomes is similar to the *flag-2 *gene cluster of O:9/2 [35]. Only parts of this cluster demonstrated low similarity to O:8/1B genes. The functionality and role of this cluster in pathogenesis are questionable, since experimental observation indicates a weak motility for O:3/4 strains *in vitro*.

Operons of the β-fimbriae usually do not resemble typical tip adhesins [36]. They may encode thin fibrillae or nonfimbrial surface structures. We found two clusters of putative beta-fimbriae in O:3/4 (Y11_14931-14971 and Y11_26051-26081), both absent from O:8/1B.

#### Chromosomally encoded type three secretion system (T3SS) and Aat-secretion

**Figure 3:**
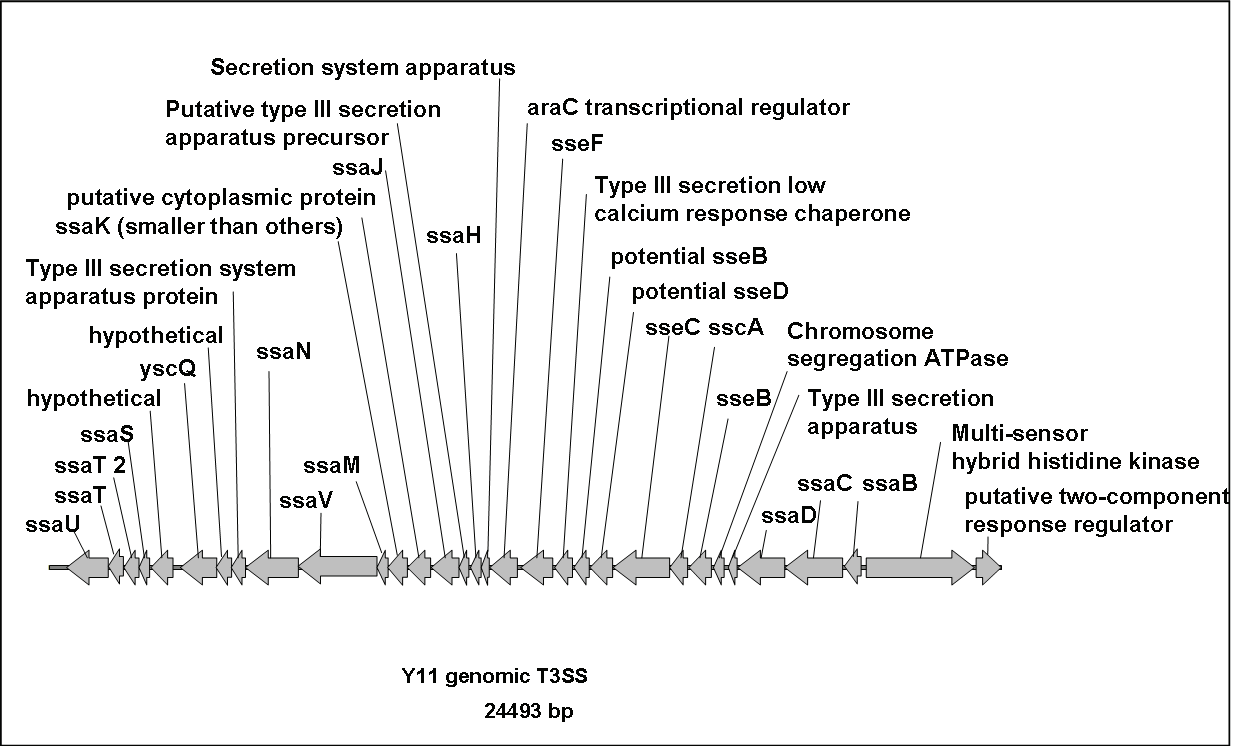
***Y. enterocolitica palearctica *O:3/4-specific chromosomally encoded type three secretion system**. This system is similar to the SPI-2 of *Salmonella*. The translocon of the apparatus is present, but SsaGH proteins of the needle are missing; also SsaR from the inner membrane ring. In addition, no effector proteins are linked directly to the secretion system gene cluster in the genome strain Y11.

The virulence-associated chromosomal *ysa *T3SS is absent in O:3/4. Even so, O:3/4 harbours an alternative 24 kbp T3SS (designated *ysp *T3SS, Y11_35171-35491, see Figure 3) in the same genome background. The *ysp *T3SS system is homologous to the *Salmonella *SPI-2 T3SS [37], but lacks some functional parts. The translocon is present, but the SsaGH proteins of the needle are missing, as well as SsaR from the inner membrane ring. In addition, there are no effector proteins linked directly to the secretion system gene cluster. Thus, the *ysp *T3SS awaits the identification of its putative effectors and possible involvement in pathogenicity or cell-cell interactions.

The *aatPABCD *cluster in enteroaggregative *E. coli *(EAEC) encodes a specialized ABC transporter, which plays a role in virulence by transporting dispersin out of bacterial cell [38]. In serobiotype O:3/4, we found a four-gene *aat *cluster with one gene homologous both to *aatB *and *aatA*. The cluster (Y11_24511 -Y11_24421) is interspaced and flanked by small hypothetical genes and transposases. The functionality in O:3/4 is yet uncharacterised, but a potential role in pathogenesis as for the EAEC, cannot be excluded.

#### Carbon source uptake and other metabolic differences

**Figure 4:**
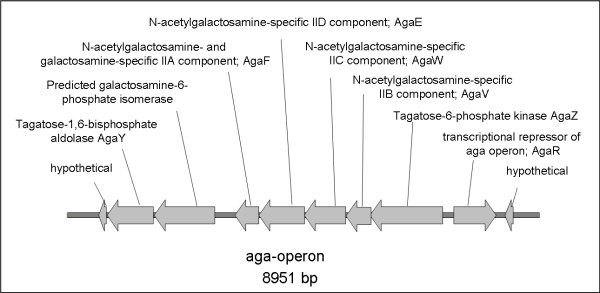
**N-acetyl-galactosamine PTS *aga*-operon (comprising *agaVWEF*) in *Y. enterocolitica *O:3/4**. Displayed genes have been subcloned into pGEM-T Easy (Promega) to supplement serobiotype O:8/1B with the additional PTS.

N-acetyl-D-galactosamine and N-acetyl-D-glucosamine are components of the intestinal mucin in pigs and humans. The amount of N-acetyl-D-galactosamine is nearly twice that of any of the other sugars present in the pig's small intestinal mucin [39]. In contrast, N-acetyl-D-glucosamine is the major amino sugar in human mucin [40]. The composition and modification of mucin is a critical defence mechanism in the prevention against pathogenic bacteria in the intestine. Enteric bacteria differ in their ability to grow on N-acetyl-galactosamine (GalNAc or Aga) and on D-galactosamine (GalN or Gam). N-acetyl-galactosamine utilized in *Yersinia *can be taken up as a carbon source by a specific phosphotransferase system (PTS). The enzymes that build the transport complex are AgaVWEF, and the genes are clustered in an operon in *Y. enterocolitica *O:3/4 (see Figure 4) that is absent in O:8/1B.

**Figure 5:**
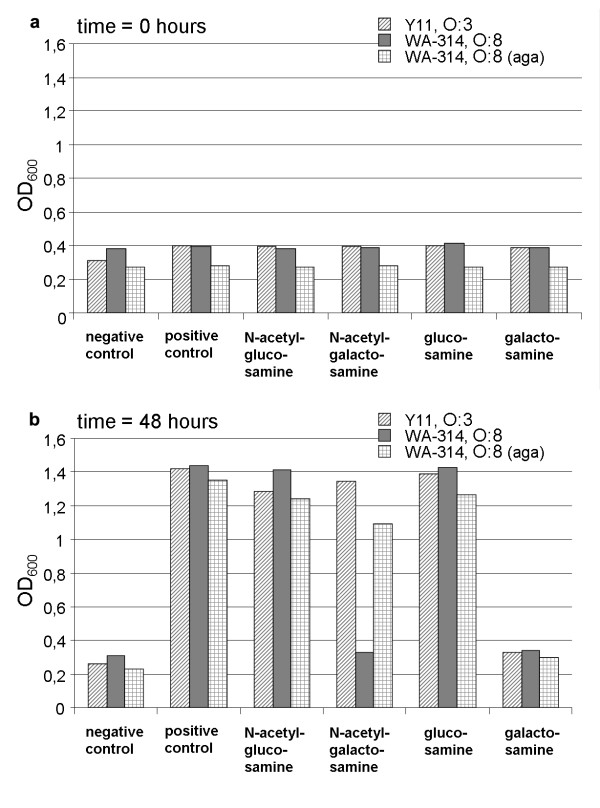
**Amino sugar growth experiment**. Strains used for the experiments are Y11 (O:3/4) and WA-314 (O:8/1B) as well as a derivative of WA-314, supplemented with the complete *aga*-operon (see text). Strains were grown overnight in M9 minimal medium with glucose, washed and inoculated in M9 containing different amino sugars and sugars as the only carbon source. Negative control was M9 without any carbon source, positive control - 0.2% glucose. a) Measurement of the optical density at 600 nm (OD_600_) directly after inoculation. b) Measurement of the optical density at 600 nm after 48 hours of growth at 27°C.

The utilisation of different amino sugars (GalNAc, Gam, GlcNAc (N-acetyl-glucosamine) and Nag (D-glucosamine)) was analysed in strains of two serobiotypes, O:3/4 and O:8/1B, in minimal medium with 0.2% amino sugars. As expected, both serobiotypes were able to grow in 0.2% N-acetyl-glucosamine and glucosamine. Since the Gam PTS encoding genes (*agaBCD*) are absent from serobiotypes O:3/4 and O:8/1B, we found all strains unable to grow in galactosamine as the only source for carbon. However, O:3/4 strain Y11 was able to use GalNAc as the only carbon source, even though we did not find a homolog for the downstream processing *agaA *gene in O:3/4 (Figure 5). To prove the sufficiency of *agaVWEF *to support growth on GalNAc, we subcloned the operon (Y11_11961-Y11_12031) into pGEM-T Easy (Promega). Strain WA-314, O:8/1B, transformed with *agaVWEF*, acquired the ability to grow in the presence of GalNAc. Thus, the *aga*-operon alone is able to support the utilisation of N-acetyl-galactosamine *in vitro *and supplies *Y. enterocolitica *with the ability to efficiently utilize this important amino sugar of the intestinal mucin.

We found a second urea transporter system in O:3/4 (Y11_22281-22341), which is independent and different from the first one shared with O:8/1B. There is no obvious explanation for the presence of two different urea clusters in O:3/4. Since the urea systems are unrelated in protein composition, they must have been acquired independently.

Many bacteria isolated from the human gastrointestinal tract show bile salt hydrolase (BSH) activity mediated by the choloylglycine hydrolase (CGH). How this enzyme contributes to the functions of bacteria in the gastrointestinal tract is not known. Studies have shown that choloylglycine hydrolase (CGH) confers the ability to resist the antimicrobial action of bile salts [41]. Therefore, the CGH may contribute to the ability of bacteria to infect the host through the oral route. We found one CGH in O:3/4 (Y11_23571). The gene for CGH is absent from O:8/1B, reflecting different niches and host infection routes.

### 4. Mobile elements shaping *Y. enterocolitica *ssp. *palearctica *genome

#### Genomic Islands

Mobile genetic elements are known to be involved in horizontal gene transfer, HGT. They utilize site-specific integrases for recombination with the core genome and use small RNA genes as attachment sites for integration. We have found 13 copies of integrase genes in the Y11 genome (strain 8081 has 21 copies), but most of them seem to be truncated and no more functional. In Y11, 5 of the 13 annotated integrase genes are located next to tRNA genes. This was also the case for a tRNA-Asn that has acquired a novel genomic island of 14.9 kbp, GIYep-01. Three different tRNA-Asn loci are found in *Yersinia*. One of these tRNA-Asn copies has acquired the HPI in O:8/1B [20], while the GIYep-01 island (Y11_15011-Y11_15121) occupies one of the tRNA-Asn copies in O:3/4. GIYep-01 has a GC content similar to the core genome sequence. In contrast, HPI has an elevated GC content and an inactivated integrase [42]. Translated CDSs of GIYep-01 show homologies to a metallo-beta-lactamase domain containing protein, SbcC, a protease like protein, an antirestriction protein and transition helper proteins, with the latter ones as typical members of mobile elements. To prove the mobility of GIYep-01 and functionality of its integrase, we have performed a nested PCR with JB470 and JB472, JB471 and JB473 primers to follow the restoration of the *attP *recombination site of the circular excised island. Results of the PCR and sequencing demonstrated the precise excision of the GIYep-1 island. Moreover, when the integrase and its attachment sites were analysed in two other serobiotype O:3/4 strains, Y8265 and Y5307, both harboured a full-length integrase and intact attachment sites.

#### Prophages as main *Y. enterocolitica ssp*. palearctica acquisitions

We found a filamentous prophage (Y11_09601-09661) in the Y11 genome that was highly homologous to CUS-1 of *E. coli *[43] and the Ypf prophage of *Y. pestis *[44]. In *Y. pestis*, the Ypf genome contains all functional modules needed for the assembly and production of viable phages and is suspected to play a role in *Y. pestis *virulence [44].

**Figure 6:**
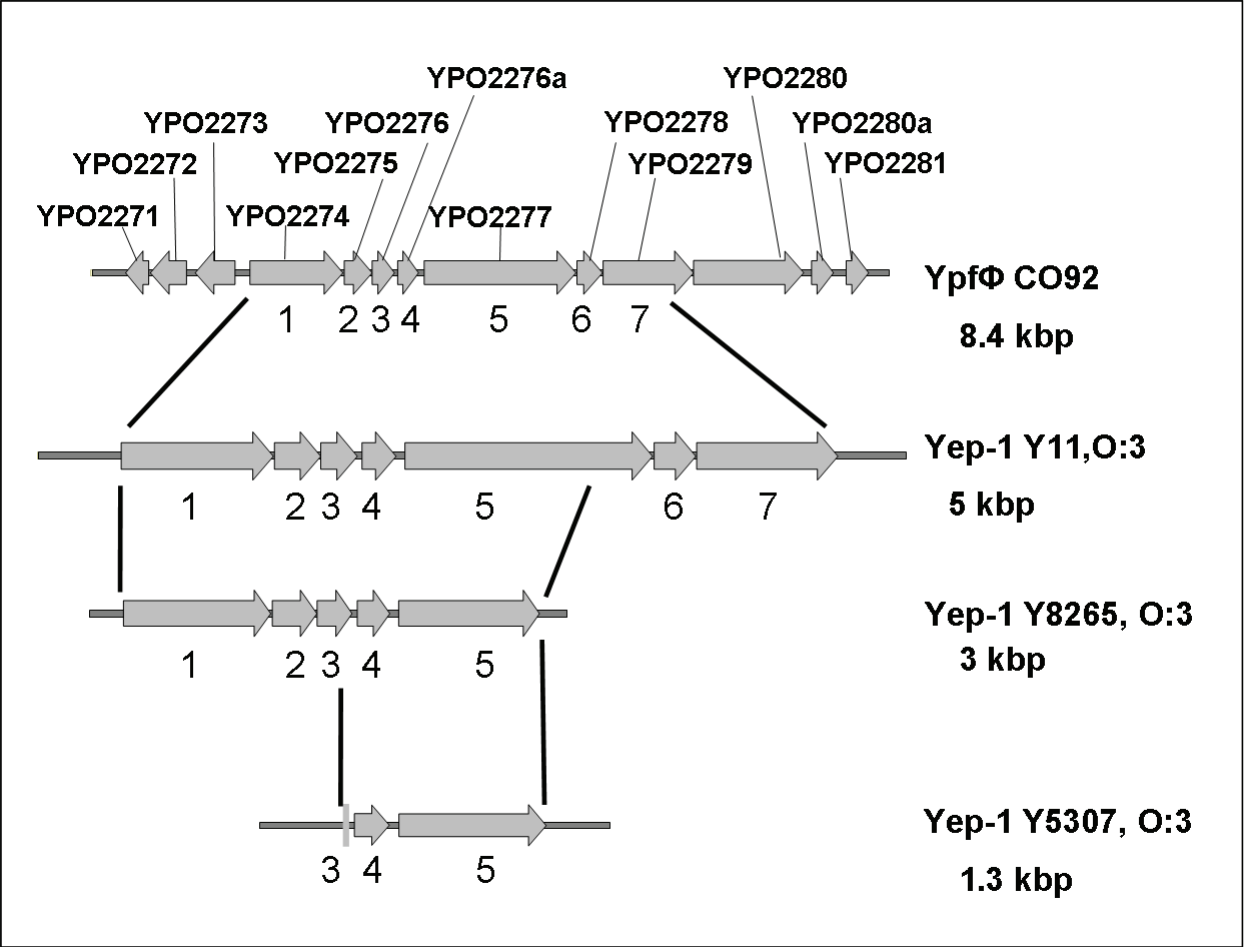
**Comparison of the *Y. pestis *Ypf prophage and the PhiYep-1 prophage in *Y. enterocolitica *O:3/4**. The Ypf prophage is representing a typical structure of a filamentous phage, with genes for morphogenesis, secretion and replication. The cluster in O:3/4 is notably reduced, and in strain Y5307 the Yep prophage is reduced to only three truncated genes left, as proven by sequencing.

The PhiYep-1 prophage is absent from strain 8081, O:8/1B; nevertheless, the infection with this prophage has been demonstrated for all three pathogenic *Yersinia *species [45]. However, the comparison of PhiYep-1 prophages in two other serobiotype O:3/4 strains, Y8265 and Y5307, showed that the prophage sequence has already suffered successive deletions in both strains (Figure 6).

We found that the PhiYep-1 prophage constitutes a part of the 28 kbp tandem repeat amplified in *Y. enterocolitica *after elevation of ampicillin levels [46]. This repeat harbours the *blaA *gene and at least part of the PhiYep-1 prophage, indicating a possible link between PhiYep-1 tandem multiplication and elevated ampicillin resistance. By PCR we proved the multiplication of the 28 kbp fragment in Y11, but also in the absence of ampicillin. Moreover, when we raised the ampicillin concentration from 100 μg/ml to 1,000 μg/ml, both for Y11 and Y5307 that lacks most of the PhiYep-1 sequence, the strains were able to grow in 1,000 μg/ml ampicillin containing LB media with similar overnight densities. Thus, the tandem multiplication of the PhiYep-1 prophage together with the nearby *blaA *region, seems not to be the only mechanism of the rapid ampicillin resistance acquisition in O:3/4.

At least two highly homologous P2-like prophages, PhiYep-2 (Y11_25141-25551) and PhiYep-3 (Y11_13081-Y11_13511) are integrated in different tRNA genes in the Y11 genome; PhiYep-2 in tRNA-Met and PhiYep-3 in tRNA-Leu. These phages are highly homologous to the P2-like prophage in *Y. pseudotuberculosis *IP32953 (additional file 4). PhiYep-3 contains a full-length integrase and was proven for its ability to leave its attachment site. The JB606 and JB608 primers designed for restoration of the 123 bp *attP *attachment site demonstrated a high frequency precise excision of PhiYep-3. Nevertheless, this prophage was absent in two other serobiotype O:3/4 strains, Y8265 and Y5307. To address dissemination of PhiYep-3 in serobiotype O:3/4 strains, we performed PCR for its presence in tRNA-Leu target site. In six out of fifteen O:3/4 strains tested, the tRNA-Leu gene was occupied by the PhiYep-3 prophage (data not shown). Thus, presence of the highly active PhiYep-3 prophage may serve as an additional epidemiological marker of recent and perhaps repeated prophage acquisitions.

#### IS-elements

IS element copy numbers in the *Yersinia *genus vary between 12 and 1,147 [47]. The number of IS elements identified in Y11 genome using the ISfinder [48] was slightly higher than that in 8081 (about 64 full length IS elements). Interestingly, the variety of IS element families was higher in O:8/1B and includes IS4 and IS200 family members that are absent from O:3/4. ISYen1 is the most frequent IS element in Y11 (more than 50 copies, see Table 2).

**Table 2:** Insertion sequence elements found in *Y. enterocolitica ssp. palearctica *Y11, O:3/4.

IS element	Number of insertion elements found in the Y11 chromosome
ISYen2A/B	7

IS1400	1

ISYen4	1

ISYal1	2

ISYen1	53

One of the ISYen1 elements located in the promoter region of *inv *effects its regulation (Frank Uliczka *et al*. 2011, manuscript accepted by PLoS Pathogens). Furthermore, many other genes found in O:3/4 in the vicinity of ISYen1 or other IS elements can influence their activity. Examples are *cbpA*, *yidE*, a formate efflux transporter and dehydrogenase, *argO*, *ycaD*, and putative virulence factors (e.g. a toxin subunit S1 precursor).

Beside the large group of ISYen1 elements, we found seven copies of the *Y. enterocolitica *ssp. *palearctica *- specific IS-element ISYen2 [49]. This IS element is related to those of the IS21 family and its two isoforms (ISYen2A/B) are present in seven genomic copies in O:3/4. A further ISYen2B copy is located in the pYVO3 virulence plasmid [49]. We have detected at least one ISYen2A/B copy also in the O:5,27/3 genome.

## Discussion

Two parallel processes, gene loss and acquisition, shape the *Y. enterocolitica *ssp. *palearctica *serobiotype O:3/4 genome. As expected, *Y. enterocolitica *ssp. *palearctica *strains do not carry the already defined high-pathogenicity associated features of 8081 O:8/1B ([50]). This decreased pathogenicity may supply the O:3/4 group with a better chance to balance its interactions with the host and support further dissemination. Instead, *Y. enterocolitica *ssp. *palearctica *demonstrates an alternative pattern of putative virulence associated determinants including an RtxA-like toxin, a dual functional insecticidal toxin, beta-fimbriae and a novel *ysp *type 3 secretion system. Since serobiotype O:3/4 has adapted to a very narrow and specific niche, the pig tonsils, the bacteriocin cluster found in O:3/4 would also provide a serious advantage in colonisation. Likewise, the ability to utilise GalNAc of the gut mucin may represent both a virulence and fitness factor of particular importance for O:3/4 and reflects its adaptation/association with its host.

The *ysp *T3SS of O:3/4 that substitutes the *ysa *T3SS of O:8/1B is analogous to T3SS systems of high-pathogenic *Y. pestis *and *Y. pseudotuberculosis*, indicating a potential role in pathogenicity. The effectors of this T3SS have to be identified. The *ysp *and *ysa *secretion systems are known to be involved both in "cross-talk" with other bacteria and with the host and they are able to support transport of "heterologous" effector molecules [51,52]. Thus, the *ysp *system might supply *Y. enterocolitica *ssp. *palearctica *with an additional advantage to subvert foreign imported effectors for its benefit even in the absence of the native ones.

Mobile genetic elements encoding multiple physiological traits play a significant role in bacterial evolution. A novel GIYep-01 genomic island that encodes a putative metallo-beta-lactamase and a protease in O:3/4 might be involved both in fitness and pathogenicity of yersiniae. For its integration, GIYep-01 utilizes a P4-like integrase like the HPI in O:8/1B. However, in contrast to the HPI that is frozen to a single tRNA-Asn site in O:8/1B, the GIYep-01 can leave its initial tRNA-Asn locus due to the activity of the functional integrase. Whether the integrase of one mobility element can affect the recombination of another one and the putative role of the GIYep-01 in *Y. enterocolitica *has still to be clarified.

The filamentous PhiYep-1 prophage of O:3/4 demonstrates a high similarity to *Y. pestis *Ypf and *E. coli *CUS-1 prophages. Both Ypf and CUS-1 are suspected to play a role in pathogenicity [43]. However, due to severe sequential deletions in the sequenced O:3/4 strains, a possible impact of this prophage on O:3/4 pathogenicity and elevated ampicillin resistance remains questionable.

Two copies of the highly similar P2-like prophages are present in the Y11 genome. PhiYep-2, the one with a truncated integrase, is frozen in tRNA-Met while PhiYep-3, harbouring an active integrase, is integrated into tRNA-Leu. Anyhow, the genetically active PhiYep-3 is present only in about 40% of the O:3/4 strains tested. Thus, the PhiYep-3 prophage seems to represent a more recent Y11 acquisition and might serve as an additional epidemiological marker for *Y. enterocolitica *ssp. *palearctica*. The coexistence and immunity to superinfection of these two closely related P2-like prophages poses an additional question to be answered.

The presence of multiple IS elements tells a story of *Y. enterocolitica *interactions with its biotic neighbours. Indeed the spectrum of IS elements differs in *Y. enterocolitica *ssp. *enterocolitica *and *Y. enterocolitica *ssp. *palearctica*, with ISYen2A/B being the low *-*pathogenicity specific insertion sequence whilst a wide variety of IS families IS3, IS4 and IS200 dominates in the high-pathogenicity group. These differences can be applied both to subspecies identification and also for tracing history of interbacterial interactions.

It is remarkable that the gene clusters with potentially closely related functions tend to occupy exactly the same positions ("hot spots") in the *Y. enterocolitica *backbone genomes (like the T3SS, the O-antigen, an AidA adhesin and haemolysin, the OspG protein kinase gene cluster, etc.). On the other hand, divergence in these clusters might result from both vertical and horizontal evolution events.

*Y. enterocolitica *ssp. *palearctica *O:3/4 has an open genome with traces of frequent gene import and wastes. These different vectors have formed the present O:3/4 genome that combines standard yersinial determinants with putative alternative virulence and fitness associated factors. Such a patchwork seems to be a prerequisite for O:3/4 successful worldwide dissemination.

## Conclusions

*Y. enterocolitica *ssp. *palearctica *O:3/4 becomes nowadays the dominating *Yersinia *worldwide. Multiple gene losses and acquisitions experienced by its genome shift this pathogen to explore an alternative patchwork of virulence and fitness determinants for the efficient proliferation. Future analysis of these combinations of different virulence traits may shed a new light on the processes and structures behind successful dissemination of bacterial pathogens in the modern world in general and of the successful emerging zoonotic pathogen *Y. enterocolitica *ssp. *palearctica *O:3/4 in particular.

## Methods

### Strains and DNA preparation

The *Y. enterocolitica *ssp. *palearctica *Y11 (DSMZ type strain no. 13030) serobiotype O:3/4 strain isolated from human stool was selected for complete genome sequencing. Strain Y8265 (derived from a human isolate, France; [53]) and Y5307 (derived from a reactive arthritis patient, institute's strain collection) of the same serobiotype O:3/4 and strain Y5,27P of serobiotype O:5,27/3 (institute's strain collection) were used for high coverage draft genome sequencing (see below). DNA was prepared using NucleoBond^® ^AXG of Macherey-Nagel (Düren, Germany) following the manufacturers instructions.

For N-acetyl-galactosamine uptake experiments, the human isolate strain WA-314 O:8/1B was used [53].

### Genome sequencing and bioinformatics

The genome sequence of *Y. enterocolitica *ssp. *palearctica *O:3/4 strain Y11 was determined by combination of high-throughput whole genome shotgun sequencing using MegaBACE (at Integrated Genomics, Jena, Germany), 454 Genome Sequencer (GS) 20 (at 454, Branford CT, USA) and an additional 454 GS FLX Titanium run (at Seq-IT, Kaiserslautern, Germany). Gaps were closed manually in cooperation with LGC Genomics (Berlin, Germany) by PCR followed by Sanger sequencing of the respective amplification products. The last gap that constituted a second copy of a highly homologous P2-like prophage was closed by primer walking on a single phage-spanning fosmid clone. Finally, the raw data were assembled using the Newbler software (454 Life Sciences Corporation, Version Software Release 2.3) into a complete genome sequence of 4,553,420 bp for the genome and an additional 72,463 bp contig representing the pYVO3 plasmid. The complete genome sequence of this strain has been published recently [17]. The draft high coverage genome sequences of three other *Y. enterocolitica ssp. palearctica *strains of human origin, Y8265 and Y5307 of serobiotype O:3/4 and Y5,27P of serobiotype O:5,27/3 were obtained in cooperation with BGI-Hongkong Co., Hong Kong. We used high-throughput Illumina sequencing technology to conduct paired-end sequencing for DNA samples, and constructed a 500 bp library with extended data of 500 Mb, and a 6 kbp library with expected data of 250 Mb. Genome assembly results in 14 large scaffolds and 215 contigs for Y8265, 18 scaffolds and 256 contigs for Y5307, and 20 scaffolds and 408 contigs for the Y5,27P strain. Genome coverage based on k-mer exceeds 105%, and genome coverage based on reads mapping was 97% for the three genomes. The genome sequences were annotated using the RAST server [54]. Genome comparisons have been done using SEED [55], the Artemis comparison tool [56], Mauve [57] and other standard comparison tools. SEED was also used to determine orthologous proteins, using the standard parameters.

Accession numbers of strains used in this study are listed in Table 3.

**Table 3:** Accession numbers of strains involved in this study

Strain name and bioserotype	Accession numbers in EMBL database
Y11, O:3/4	FR729477 and FR745874 (plasmid)

8081, O:8/1B	NC_008800 and NC_008791 (plasmid)

105.5R(r), O:9/3	CP002246 and CP002247 (plasmid)

W22703, O:9/2	AF102990 (plasmid)

Y8265, O:3/4	CACU01000001-CACU01000014

Y5307, O:3/4	CACV01000001-CACV01000018

Y5,27P, O:5,27/3	CACW01000001-CACW01000020

### Excision of genetic mobile elements

The excision of GIYep-01 island was verified by nested PCR amplifying the *attP *attachment sites of the circular excised element (the second PCR covering about 420 bp) followed by subsequent sequencing. The following oligonucleotides have been used (bold letters, reverse orientation oligonucleotides): JB470, agaatcggaaactttgaatggttt, JB472, CACATCAGGCACTTCTCCAGG, and JB 471, ttgagccgttaagagacatttgg, and JB473, TTAACAGAAATAGCGCCCAT.

In the case of the PhiYep-3 excision, a single PCR amplification step was sufficient for amplification of the *attP *attachment site of the circular excised prophage (about 500 bp, proven by subsequent sequencing of the PCR product). For PhiYep-3, the following oligonucleotides have been used: JB606, GGCGTGTTGTGGATGTAAT and JB608, atgtcagtatatttggcgat. For PhiYep-3 dissemination analysis, 15 strains of serobiotype O:3/4 have been tested.

### N-acetyl-galactosamine experiments

The 8.4 kbp *aga*-operon (Y11_11961-Y11_12031) has been subcloned into pGEM-T Easy (Promega) using the following oligonucleotides: JB506 cagcgtcgtacttgatgatttgc and JB507 ATCATCTGTTGGGCGACACG. The *aga*-supplemented serobiotype O:8/1B strain was grown in the presence of carbenicillin (300 μg/ml) to maintain the plasmid, serobiotype O:3/4 and O:8/1B wild type strains were cultivated without antibiotics. M9 minimal medium supplemented with 1 mM MgSO_4 _and 0.1 mM CaCl_2 _and the appropriate amino sugars (0.2% of N-acetyl-galactosamine, N-acetyl-glucosamine, glucose and galactose) was used in all experiments. Tryptophan (Trp) was added (200 μg/ml) to support sufficient *Yersinia enterocolitica *growth. The bacteria were grown in 0.01 M glucose supplemented M9 overnight (16-20 hours) as a preculture, pelleted and washed with M9 without sugar additives. The optical density was measured and all samples were inoculated to the same OD_600 _0.2-0.3. Experiments were carried out in 10-20 ml M9 in 50 ml Falcon tubes. The optical density of the cultures was measured after inoculation and 48 hours.

## Further particulars

The authors declare that they have no competing interests.

## Authors' contributions

JB has carried out the main genome comparisons, designed and executed all experiments, participated in the gap closure of the genome sequence and revised the sequence. JB also wrote major parts of the manuscript and designed figures and tables. UA has done further gene comparisons, gave setup advice for the sugar uptake experiments and participated in the composition of the manuscript. DH participated in the genome sequence alignment, conducted the quality control of the assembled raw data and added helpful ideas to the manuscript. JH and AR have planned and supervised the experiments and genome comparisons, and AR has written major parts of the introduction and discussion. All authors read and approved the final manuscript.

## Supplementary Material

Additional file 1 **Additional table with general CDS features of serobiotypes O:3/4 and O:8/1B**. Click here for file

Additional file 2 **Additional Excel table with all protein comparisons (including locus tags) of the annotated genome of strain Y11 with related genomes (serobiotype O:5,27/3; two additional serobiotype O:3/4 strains; serobiotype O:8/1B strain 8081)**. Click here for file

Additional file 3 **Additional figure with a comparison of the pYV plasmid of serobiotypes O:3/4, O:9/2 (pYVe227) and O:8/1B using the Artemis Comparison Tool (ACT)**. Click here for file

Additional file 4 **Additional figure with a comparison of the genome sequence of the two P2-like prophages PhiYep-2 and PhiYep-3 with the homologous P2-like prophage in *Y. pseudotuberculosis *IP 32953**. Click here for file
