# Supplementary material for: Yersinia enterocolitica palearctica serobiotype O:3/4 - a successful group of emerging zoonotic pathogens
Source: BMC Genomics. 2011 Jul 6;12:348. doi: 10.1186/1471-2164-12-348 (PMC3161016; doi:10.1186/1471-2164-12-348)
Supplement: Additional file 1 — Additional table with general CDS features of serobiotypes O:3/4 and O:8/1B. [file 1471-2164-12-348-S1.PDF]

Additional file 1
Batzilla *et al.*, 2011
Yersinia enterocolitica palearctica O:3/4 – a successful group of emerging zoonotic pathogens.

	**Strain Y11**	**Strain 8081**
Number of annotated CDSs	4,355	4,243
Number of hypothetical CDSs	623 (plus 18 on the pYV plasmid)	485 (plus 7 on the pYV plasmid)
Y11 specific CDSs	629 (363 of them are hypothetical)	-
8081 specific CDS	-	592 (249 of them are hypothetical)

Analyses have been performed using the RAST annotated genomes and the SEED comparison tools with the standard parameters. Only those CDSs which were annotated as “hypothetical protein” or were predicted but left blank in the field “function” were regarded as hypothetical. SEED calculated CDSs with identities below 40% have been assumed to be strain specific.
