# Supplementary material for: Yersinia enterocolitica palearctica serobiotype O:3/4 - a successful group of emerging zoonotic pathogens
Source: BMC Genomics. 2011 Jul 6;12:348. doi: 10.1186/1471-2164-12-348 (PMC3161016; doi:10.1186/1471-2164-12-348)
Supplement: Additional file 3 — Additional figure with a comparison of the pYV plasmid of serobiotypes O:3/4, O:9/2 (pYVe227) and O:8/1B using the Artemis Comparison Tool (ACT). [file 1471-2164-12-348-S3.PDF]

**Additional file 3**
Batzilla *et al*., 2011
*Yersinia enterocolitica palearctica* O:3/4 – a successful group of emerging zoonotic pathogens.

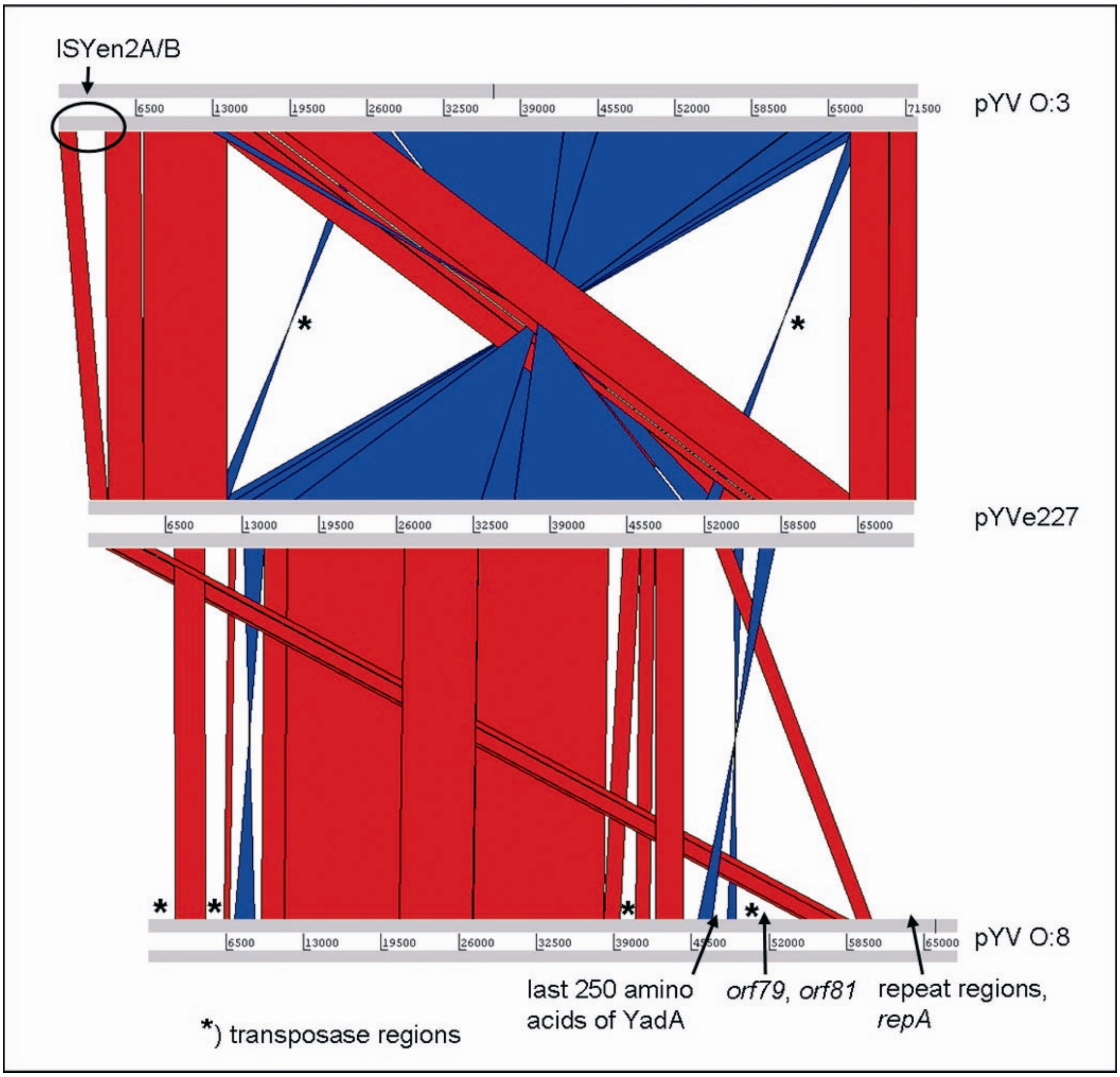


Comparison of the pYV plasmid of serogroups O:3, O:9 (pYVe227) and O:8 using the Artemis Comparison Tool (ACT). In red, regions found likewise in the different plasmids; in blue, regions found inverted in the different serogroups. Regions without colour coded connections are unique (homologous regions smaller than 500bp were, for simplicity purposes, not included into the graphic), mostly transposase regions, IS elements and hypothetical CDS.
