# Supplementary material for: Yersinia enterocolitica palearctica serobiotype O:3/4 - a successful group of emerging zoonotic pathogens
Source: BMC Genomics. 2011 Jul 6;12:348. doi: 10.1186/1471-2164-12-348 (PMC3161016; doi:10.1186/1471-2164-12-348)
Supplement: Additional file 4 — Additional figure with a comparison of the genome sequence of the two P2-like prophages PhiYep-2 and PhiYep-3 with the homologous P2-like prophage in Y. pseudotuberculosis IP 32953. [file 1471-2164-12-348-S4.PDF]

Additional file 4
Batzilla *et al*., 2011
Yersinia enterocolitica palearctica O:3/4 – a successful group of emerging zoonotic pathogens.

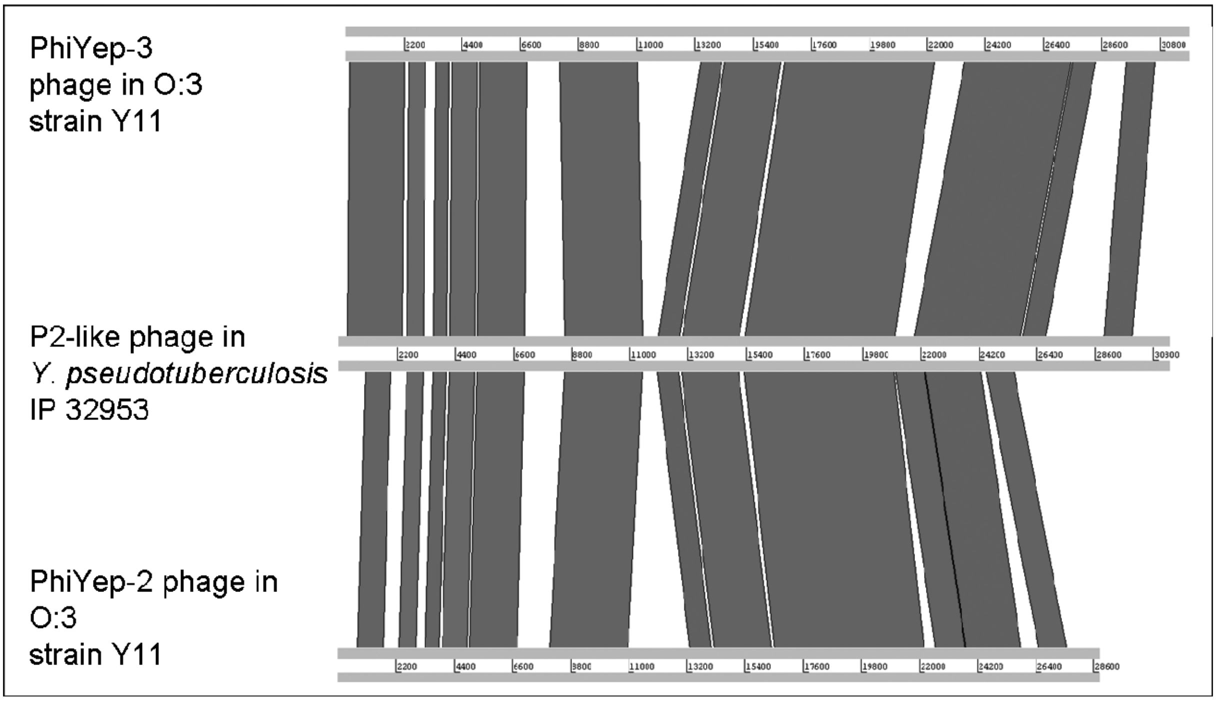

Comparison of the genome sequence of the two P2-like prophages PhiYep-2 and PhiYep-3 with the homologous P2-like prophage in *Y. pseudotuberculosis* IP 32953. The three prophages are highly homologous, but only for Yep-3 a complete integrase and the excision of the prophage has been proven. Yep-2 has a reduced size compared to Yep-3 and the integrase is truncated.
